# Integrated transcriptomics and metabolomics analysis of volatiles and variations in carotenoid biosynthesis during different developmental stages of *Camellia huana*

**DOI:** 10.1186/s12870-025-06549-z

**Published:** 2025-05-31

**Authors:** Zhiping Chen, Yongxin Zhou, Xia Jiang, He Li, Xiaohui Liu, Lunxiu Deng, Gang Wang

**Affiliations:** 1 Guizhou Academy of Forestry, Guiyang, Guizhou 550005 China; 2 Key Laboratory of National Forestry and Grassland Administration on Biodiversity Conservation in Karst Mountainous Areas of Southwestern, Guiyang, Guizhou 550005 China; 3 Guizhou Liping Rocky Desertification Ecosystem National Observation and Research Station, Qiandongnan Prefecture, 556200 China; 4 Guizhou University of Traditional Chinese Medicine, Guiyang, Guizhou 550025 China

**Keywords:** *Camellia huana*, Petals, Transcriptomics, Metabolomics, Carotenoids

## Abstract

**Background:**

*Camellia huana*, an endangered plant species with a high ornamental and medicinal value, is designated as a Class I protected plant in China. In the natural state, the flowers of *C. huana* have golden color and fragrance; however, our study group found that the outer layer of the petals of one *C. huana* plant was mutated and showed red color. Plant petals show a variety colors due to the synthesis and accumulation of pigments. However, thus far, few studies have investigated the formation and differences in flower color in *C. huana*. Carotenoids are the main color-forming components in *C. huana* flowers. *C. huana* petals also contain volatile compounds that impart a distinct fragrance to the flowers. In the present study, to better understand the regulatory mechanisms of carotenoids and volatile compounds in the petals of *C. huana* flowers of different colors, we used flowers obtained at different developmental periods of *C. huana* as the experimental materials and conducted metabolome and transcriptome analyses.

**Results:**

The results showed that *C. huana* is rich in volatile compounds. A total of 372 metabolites were detected in *C. huana*, which mainly included 72 terpenoids, 67 heterocyclic compounds, nitrogen-containing compounds, esters, aromatic hydrocarbons, 39 alcohols, and others; terpenoids, heterocyclic compounds, and esters were the predominant volatiles. Forty carotenoids were identified by carotenoid content analysis, and 10 genes were involved in carotenoid biosynthesis were screened for their significant differential expression, namely 15-cis-phytoene synthase (*crtB*), prolycopene isomerase (*crtISO*, *crtH*), beta-carotene 3-hydroxylase (*crtZ*), beta-carotene isomerase (*DWARF27*), 9-cis-beta-carotene 9ʹ,10ʹ-cleaving dioxygenase (*CCD7*), zeaxanthin epoxidase (*ZEP*, *ABA1*), 9-cis-epoxycarotenoid dioxygenase (*NCED*), xanthoxin dehydrogenase (*ABA2*), abscisate beta-glucosyltransferase (*AOG*), and (+)-abscisic acid 8ʹ-hydroxylase (*CYP707A*). A total of 12,089 differential genes were screened by transcriptome analysis.

**Conclusions:**

The results of this study enriched the transcriptome data and provided new insights into the mechanisms of color and odor formation in the flowers of *C. huana*.

**Supplementary Information:**

The online version contains supplementary material available at 10.1186/s12870-025-06549-z.

## Introduction

*Camellia huana* T. L. Ming et W. J. Zhang is a small shrub with golden flowers, and it belongs to the genus *Camellia* in the Theaceae family. This plant is mainly distributed in southern Guizhou and northern Guangxi in China [1]. It has the highest natural distribution latitude among *Camellia* species in China, with a narrow distribution range. *C. huana* has been included in the Red List of Endangered Species in 2015. This plant is widely known as “tea queen,” “plant giant panda,” and “plant living fossil” [2, 3]. *C. huana* has both medicinal and edible properties. Modern pharmacological studies have shown that *C. huana* contains flavonoids [4], polysaccharides [5], saponins [6], amino acids [7], terpenoids [8], and other bioactive components. *C. huana* extract exhibits antitumor, anti-inflammatory, and anti-oxidative properties [9, 10].

Flowers are generally regarded as the essence of plants, and they contain a wide variety of metabolites such as flavonoids, terpenoids, carotenoids, and anthocyanins [11]. *C. huana* develops fragrant flowers containing volatile compounds. A previous study identified 73 types of volatile compounds in *C. huana* flowers [12]. Primarily alcohols, aldehydes, and C6 volatile compounds were found to significantly contribute to the volatile components of *C. huana* petals. Luo et al. [13] performed headspace solid-phase microextraction mass spectroscopy and discovered that the content of pyrazine compounds in *C. japonica* was higher than that of other compounds. Yao et al. [14] used the UPLC-C-Q-Orbitrap HRMS combined database and identified 180 chemical components from *C. huana*, including 28 flavonoids, 9 phenylpropanoid compounds, 15 terpenoids, and other compounds. In another study, headspace solid phase microextraction and gas chromatography and mass spectrometry were used to analyze the volatile compounds and their relative contents in *C. huana* flowers; 237 types of volatile compounds were isolated and identified, which accounted for 99.58% of the total volatile compounds [15].

Tea contains 54 different types of volatile compounds [16]. Among these compounds, terpenoids exhibit various effects on plants, including allelopathic effects on plants [17], antibacterial and bactericidal effects on microorganisms [18], toxic effects on herbivorous insects [19], and supportive effects on flower pollination [20]. Furthermore, the enhanced sweetness of citrus flowers can be attributed to the increased presence of terpene tetraterpenols [21]. Additionally, terpenoids can lead to an overall increase in the quantity and diversity of volatiles in plants [22].

Color is an important biological characteristic of plants and the most adaptive phenotypic trait in the natural evolution of plants [23]. Carotenoids are natural pigments and critical color-forming compounds that play an important role in color formation in flowers and fruits. They are one of the main compounds that cause plants to change color to yellow and red [24]. Carotenoids can exert several physiological effects such as photoprotective, antioxidative, and anti-stress effects [25]. They are also the synthetic precursors of phytohormones such as abscisic acid, strigolactone, and carrot fat and participate in the specific regulation of related genes and proteins, which play a crucial role in the growth and development of tea plants [26].

Some studies have shown that flavonoids [27] and carotenoids [28] are closely associated with color formation in *Camellia* flowers; hence, it is pivotal to study the synthesis of carotenoids to improve the economic value of *Camellia*. Functional characterization of the genome and transcriptome data of *Lonicera japonica* showed that carotenoid cleavage dioxygenases (LjCCD 4 and LjCCD 1b) play an important role in flower coloration by cleaving lutein, β-carotene, and 100-dehydroxy-β-carotene aldehyde to produce colorless carotenoid (C27-dehydroxy-carotenoid and C14-dialdehyde) and volatile C13-dehydroxy-carotenoid [29]. The analysis of differentially expressed genes (DEGs) and the co-expression network showed that carotenoid synthesis is mainly regulated at the transcriptional level; moreover, phytoene synthase, β-carotene 3-hydroxylase (CrtZ), and capsicum red synthase (CCS1) play a synergistic role in carotenoid synthesis [30]. Thus far, multi-omics studies conducted on tea plants mainly included genomics, transcriptomics, and metabolomics analyses. However, volatile metabolomics and carotenoid metabolomics of *C. huana* have been rarely investigated [31, 32]. In the present study, we integrated transcriptome and metabolome analyses to determine volatile metabolites of *C. huana* at different developmental stages and assessed differences in gene expression and metabolomics in *Camellia* in different developmental periods to provide insights for further protection and use of *C. huana*.

## Materials and methods

### Plant materials

**Fig. 1:**
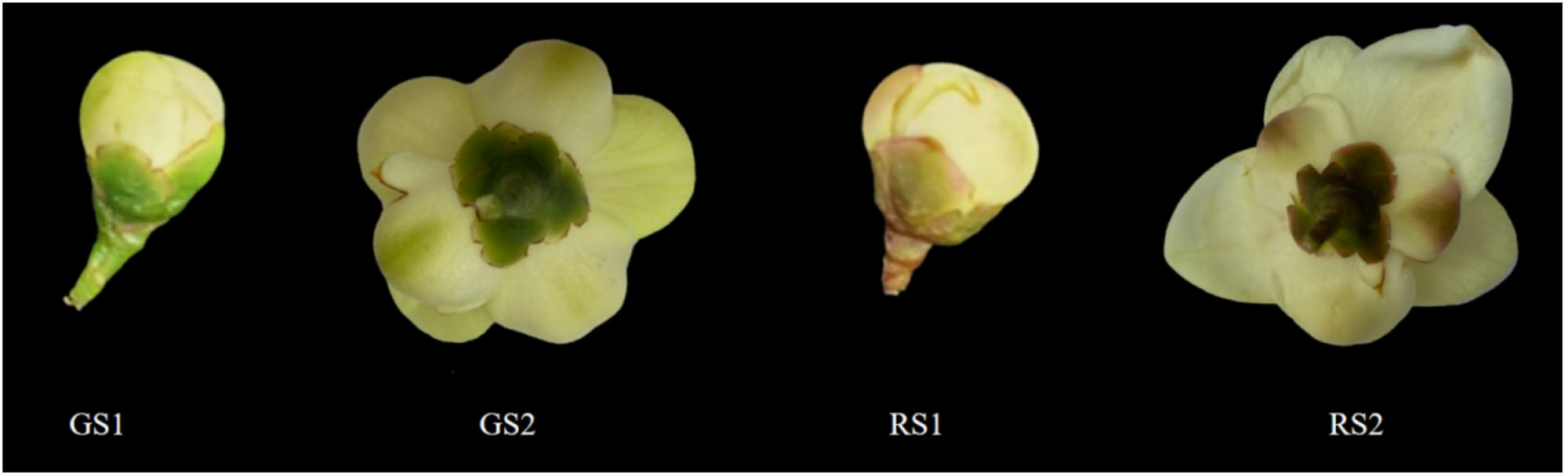
Images of different phenotypes of *C. huana*

The experimental materials (*C. huana* flowers) were collected from the Guizhou Forestry Science Research Institute (N 22°49′11′′, E 108°20′53′′). The flowers were golden yellow in color in their natural growth state; additionally, our study group found that one *C. huana* plant showed a color change in the outer petal layer, which appeared red. The golden yellow flowers collected in the bud stage and full bloom stage were designated GS1 and GS2, respectively, and the flowers with the red outer petal layer collected in the bud stage and full bloom stage were designated RS1 and RS2, respectively, as shown in Fig. 1. Twelve flower samples were collected and assigned to four groups, with three samples in each group. The samples were sorted and packaged, frozen in liquid nitrogen, and stored at -80 °C for transcriptome sequencing and extraction of volatile compounds and carotenoid metabolites.

### Targeted determination of carotenoids by LC-MS/MS

#### Sample pretreatment

The cryopreserved *C. huana* flowers were subjected to ball mill grinding (30 Hz, 1 min) and ground to form a powder. Next, 50 mg of the ground sample was weighed and subjected to extraction with 0.5 mL of hexane/acetone/ethanol mixture (1:1:1, v/v/v) containing 0.01% 2,6-Di-tert-butyl-4-methylphenol (g/mL). The obtained extract was vortexed for 20 min at room temperature and then centrifuged at 12,000 r/min for 5 min 4 °C; subsequently, the supernatant was collected. The extraction and centrifugation processes were repeated once, and the obtained supernatants were combined. The extract was concentrated and redissolved in 100 µL of methanol/methyl tert-butyl ether mixture (1:1, v/v), filtered through a 0.22 μm membrane filter, and stored in an amber-colored injection bottle for liquid chromatography-tandem mass spectrometry (LC-MS/MS) analysis.

#### Chromatography and MS acquisition conditions

The data acquisition system mainly included an ultra-performance liquid chromatography (UPLC) system (ExionLC TM AD, https://sciex.com.cn/) and a tandem mass spectrometry (MS/MS) system (QTRAP^®^ 6500plus, https://sciex.com.cn/). The main liquid phase conditions were as follows: column: YMC C30 (3 μm, 100 mm × 2.0 mm i.d.); mobile phase A: methanol/acetonitrile (1:3, v/v) with 0.01% BHT and 0.1% formic acid; mobile phase B: methyl tertiary butyl ether with 0.01% BHT; gradient elution program: 0 min and 3 min for 100:0 (v/v) for A/B, 30:70 (v/v) at 5 min, 5:95 (v/v) at 9 min, and 100:0 (v/v) at 10 and 11 min; flow rate: 0.8 mL/min. The column temperature was 28 °C, with an injection volume of 2 µL. The MS conditions were as follows: atmospheric pressure chemical ionization source temperature, 350 °C; air curtain gas, 25 psi.

### Determination of target volatiles by GC-MS/MS

#### Sample extraction process

The samples were stored at -80 °C after the removal of impurities. After grinding with liquid nitrogen and adequate vortex mixing, 500 mg of each sample was weighed and placed in a headspace vial. Next, a saturated NaCl solution and 10 µL (50 ug/mL) of an internal standard solution were added to the samples, and extraction was performed using an automatic headspace solid-phase microextraction system (HS-SPME). The obtained extract was then analyzed by gas chromatography-mass spectrometry (GC-MS).

#### Chromatography and MS acquisition conditions

HS-SPME extraction was performed under the following conditions: 60 °C, shaking for 5 min, headspace extraction for 15 min, and desorption at 250 °C for 5 min; this was followed by chromatographic separation and identification by GC-MS. The chromatographic conditions were as follows: DB-5MS capillary column (30 m × 0.25 mm × 0.25 μm, Agilent J & W Scientific, Folsom, CA, USA), high purity He (purity > 99.999%) as the carrier gas, constant flow rate of 1.2 mL/min, inlet temperature of 250 °C, nonsplit injection, and solvent delay of 3.5 min. The heating program was as follows: maintaining temperature initially at 40 °C for 3.5 min, followed by warming up to 100 °C at the rate of 10 °C/min, then to 180 °C at the rate of 7 °C/min, and finally to 280 °C at the rate of 25 °C/min for 5 min. The MS conditions were as follows: electron bombardment ion source temperature, 230 °C; quadrupole temperature, 150 °C; MS interface temperature, 280 °C; electron energy, 70 eV; and scanning mode, selected ion detection mode.

### Transcriptome sequencing

#### RNA extraction, cDNA library Preparation and RNA sequencing

Four libraries were constructed for transcriptome sequencing based on samples of *C. huana* flower representing two flower colors and two different developmental stages. First, sample RNA was extracted by TRIzol reagent (TaKaRa, Da-lian, China) according to the instructions, and then the integrity of RNA and DNA contamination were analyzed by agar-gel electrophoresis. Total RNA was then identified and quantified using Qubit 4.0 fluorometer /MD enzyme marker, Nano Drop and Agilent 2100 bioanalyzer (Thermo Fisher Scientific, MA, USA). Finally, the library is constructed, and on-machine sequencing is carried out after qualified library inspection; after qualified library inspection, different libraries are carried out pooling according to the target on-machine data amount, sequenced by Illumina high-throughput sequencing platform, and 150 bp paired end reading is generated.

#### Analysis of differentially expressed genes (DEGs) and functional annotations

To ensure the quality of Reads, fastp software was used to filter the original data and remove the connectors. reads with N content exceeding 10% of the reads base number and low-quality bases (Q ≤ 20) were sequenced to obtain Clean Reads [33]。Clean Reads data were sequentially compared with *C. oleifera* genome [34]using HISAT2 software [35]. DESeq2 was used to analyze the differential expression between the sample groups [36]。Set the filtering criterion to the difference multiple. The difference multiple|log2FoldChange| > 1 and FDR < 0.05 were used to screen differential genes (DEGs). Identify the differences of metabolites using Kyoto encyclopedia (KEGG, https://www.genome.jp/kegg) gene and genome metabolism library compare and comment, for eligible differentially expressed genes KEGG Pathway. Venn diagrams of differential metabolites were plotted using TBtools.Transcriptome data were analyzed by the KEGG pathway enrichment analysis and the volcano heatmap by using R software.

## Results and discussion

### Metabolomics analysis of volatile substances in *C. huana*

**Fig. 2:**
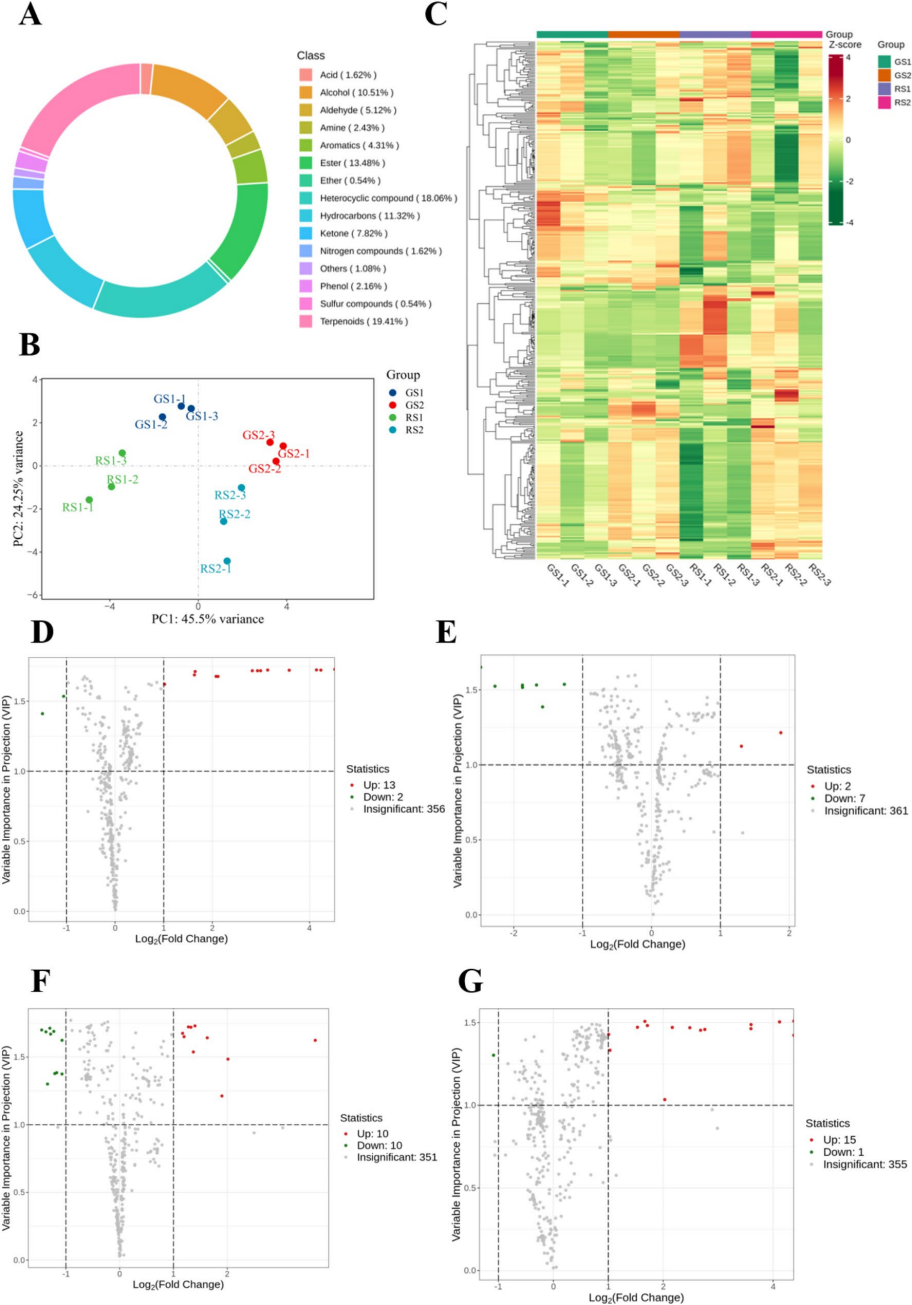
(**A**) Classification and proportion of volatile organic compounds (VOCs) detected in flowers of *C. huana*(**B**) Principal component analysis (PCA)(**C**) Hierarchical clustering heatmap of VOCs accumulation of *C. huana* flowers Volcano plots of different pairs of groups. (**D**) GS2 vs. GS1 (**E**) RS1 vs. GS1 (**F**) RS2 vs. GS2 and (**G**) RS2 vsRS1. The green dots represent down-accumulated VOCS, and the red dots represent up-accumulated VOCS

The GC-MS total ion chromatograms (TICs) of the mixed samples and the quantity control (QC) samples were compared to initially examine the reproducibility of the analytical method. As shown in Supplementary Fig. 1. The total ion flow curves for metabolite detection overlapped well, i.e., the retention times and peaks and the corresponding intensities were consistent. The entire processing method and the instrumental analysis system were stable and reliable for subsequent analyses. The GC-MS detection platform and the self-constructed database were used to study the volatile metabolites during the development of different flower colors of *C. huana*. A total of 372 metabolites were detected, which included 15 classes of compounds: 72 terpenoids, 67 heterocyclic compounds, 9 amines, 39 alcohols, 16 aromatics, 8 phenols, 6 nitrogen-containing compounds, 2 ethers, 19 aldehydes, 6 acids, 42 hydrocarbons, 29 ketones, 50 esters, and 4 other compounds. A total of 371, 372, 370, and 372 volatile organic compounds (VOCs) were detected in GS1, GS2, RS1, and RS2, respectively, and 370 VOCs were detected in all samples. The category with the highest number of VOCs was terpenes (72, 19.41%), followed by heterocyclic compounds (67, 18.06%), and esters (50, 13.48%) (Fig. 2A). These three groups are considered the main components of the floral fragrance of *C. huana*. Among the terpenes, α-stigmasterol was not detected at the bud stage of flowers of different colors, and cis-chrysanthemum alcohol was not detected at the bloom stage when the flower color was red.

Principal component analysis (PCA) of the 372 VOCs from the four *C. huana* materials showed a tendency for the metabolome groups to separate among the four groups, thus suggesting differences in the contents of VOCs in flowers collected at different developmental stages. The analysis revealed two principal components, wherein the first and second principal components (PC1 and PC2) explained 45.5% and 24.25% of the features of the original data, respectively, with a cumulative contribution of 69.75%. The PC1 component differentiated GS1 and RS1 from GS2 and RS2. The results showed a clear trend of metabolite separation between the groups and good biological reproducibility within the groups; this finding was consistent with the grouping information and could be used for the subsequent analysis of differential metabolites (Fig. 2B). Hierarchical cluster analysis (Fig. 2C) showed a good correlation between each sample of this study.

### Differential VOCs in the different stages of the flowering process

To elucidate the volatile metabolites present during the growth process of *C. huana* from Guizhou, the main differences between these processes were analyzed. According to the screening criteria for differential metabolites, 32 differential metabolites were screened, most of which were terpenoids. Flowers at the different developmental stages from GS2 vs. GS1, RS1 vs. GS1, RS2 vs. GS2, RS2 vs. RS1, and other comparison groups were compared and screened. Fifteen differential metabolites (including 13 upregulated compounds and 2 downregulated compounds) were detected between GS2 and GS1, 9 differential metabolites (including 2 upregulated compounds and 7 downregulated compounds) between RS1 and GS1, 20 differential metabolites (including 10 upregulated compounds and 10 downregulated compounds) between RS1 and GS2, and 16 differential metabolites (including 15 upregulated compounds and 1 downregulated compound) between RS2 and RS1 (Figs. 2D-G) (Supplementary Table 1). These results suggest that VOCs accumulate in large quantities during the development and maturation of *C. huana*.

**Fig. 3:**
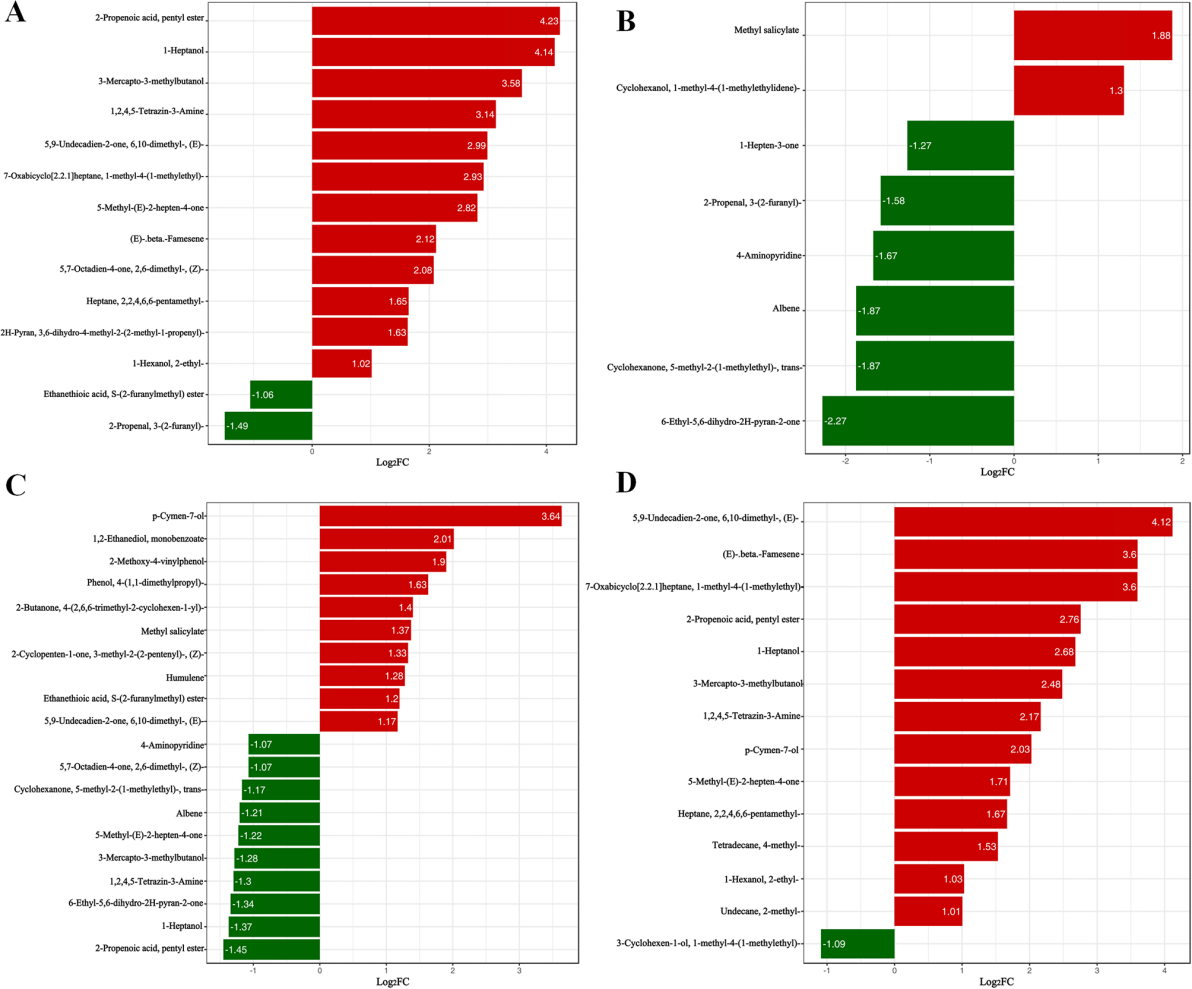
Differential metabolite bars with up-regulated metabolites in red and down regulated metabolites in green (**A**) GS2 vs. GS1 (**B**) RS1 vs. GS1 (**C**) RS2 vs. GS2 and (**D**) RS2 vsRS1

The differential expression of VOCs may be an important factor that affects aroma emission during *C. huana* maturation. Therefore, we focused on analyzing the VOCs that were significantly upregulated or downregulated. The differential fold change in metabolite expression in each group was compared in combination with the grouping of specific samples, and the top 20 metabolites were selected to plot differential metabolic bar charts (Fig. 3).GS2 compared to GS1, 2-propenoic acid, pentyl ester, (E)-β-famesene, 7-oxabicyclo[2,2,1]heptane, 1-methyl-4-(1-methylethyl)-, 2 H-pyran, 3,6-dihydro-4-methyl-2-(2-methyl-1-propenyl)-, 5,7-octadien-4-one, 2,6-dimethyl-, (Z)- were increased by 4.23-fold, 2.21-fold, 2.93-fold, 1.63-fold, and 2.08-fold, respectively. RS1 compared to GS1, the content of methyl salicylate, cyclohexanol, and 1-methyl-4-(1-methylethylidene)- increased by 1.88-fold and 1.3-fold, while that 6-ethyl-5,6-dihydro-2 H-pyran-2-one decreased by 2.27-fold. Furthermore, compared to GS2, the contents of p-cymen-7-ol, 1,2-ethanediol, and monobenzoate were reduced by 3.64-fold and 2.91-fold, respectively, in RS2. 5,9-Undecadien-2-one, 6,10-dimethyl-, (E)- in RS2 compared to RS1, (E)-β-famesene, 7-oxabicyclo[2,2,1]heptane, 1-methyl-4-(1-methylethyl)-increased 4.12-fold, 3.6-fold, and 3.6-fold, respectively.

**Fig. 4:**
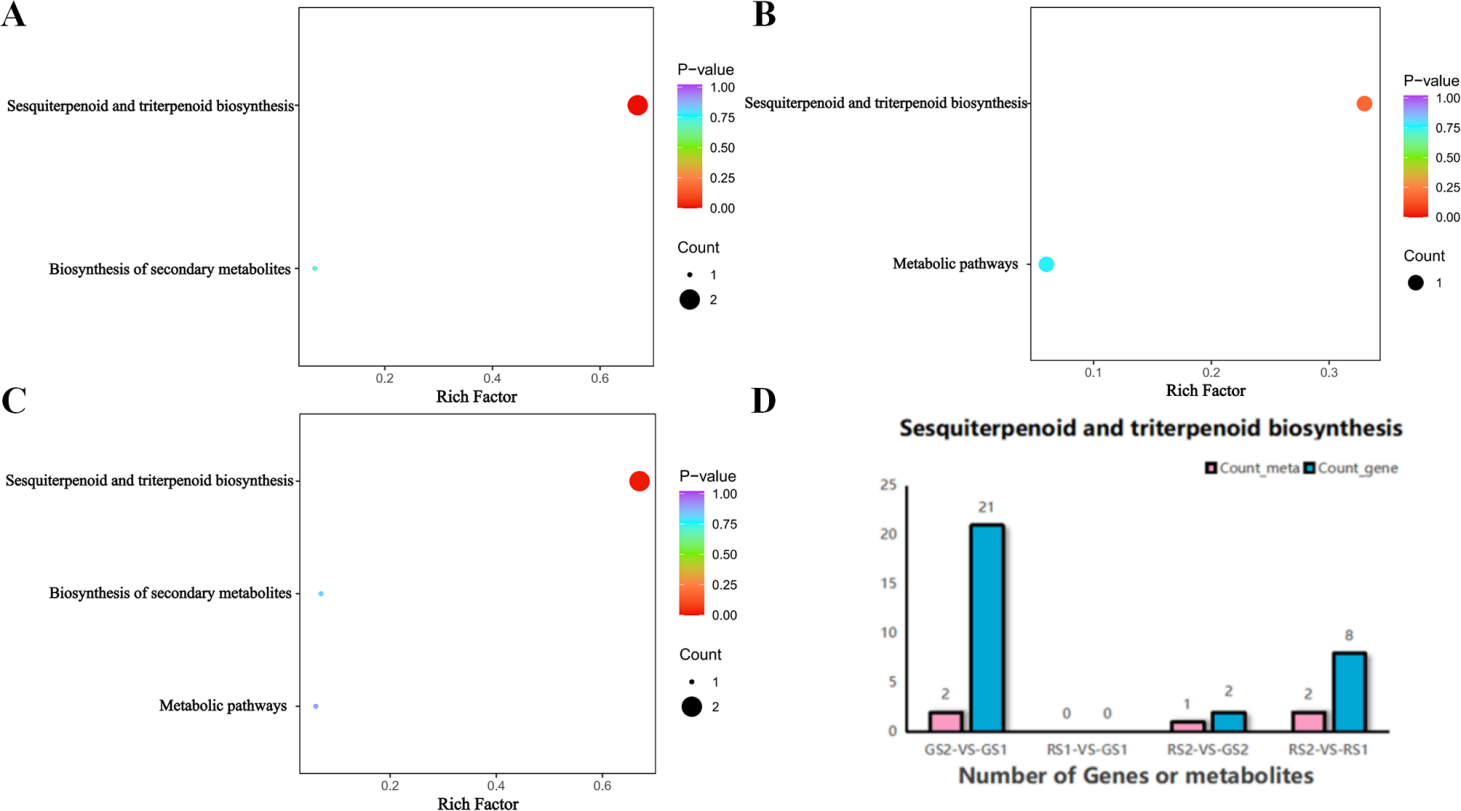
**A-D**. KEGG enrichment pathway maps for differential metabolites representing GS2 vs. GS1, RS2 vs. GS2, and RS2 vs. RS1, respectively. F. KEGG pathway transcription and metabolism histograms (histograms were plotted using the two sets of enriched KEGG pathways and show the number of differential metabolites and DEGs enriched in a pathway). To elucidate the dynamics of carotenoid metabolite content during the growth and development of *C. huana* from Guizhou, LC-MS/MS analysis was performed at various developmental stages, according to the screening criteria for differential metabolites (VIP > 1.0, fold change ≥ 2, and fold change ≤ 0.5)

The KEGG database was further used to analyze the enrichment pathways of metabolites with significantly differential expression at the different developmental stages of *C. huana*. The predominant enriched pathways were biosynthesis of sesquiterpenes and triterpenoids, biosynthesis of secondary metabolites, and biosynthesis of metabolic pathways. As shown in Fig. 4 (A-D), the most significantly enriched pathway was the biosynthesis pathway of sesquiterpenes and triterpenoids (ko00909, *P* < 0.05).

**Fig. 5:**
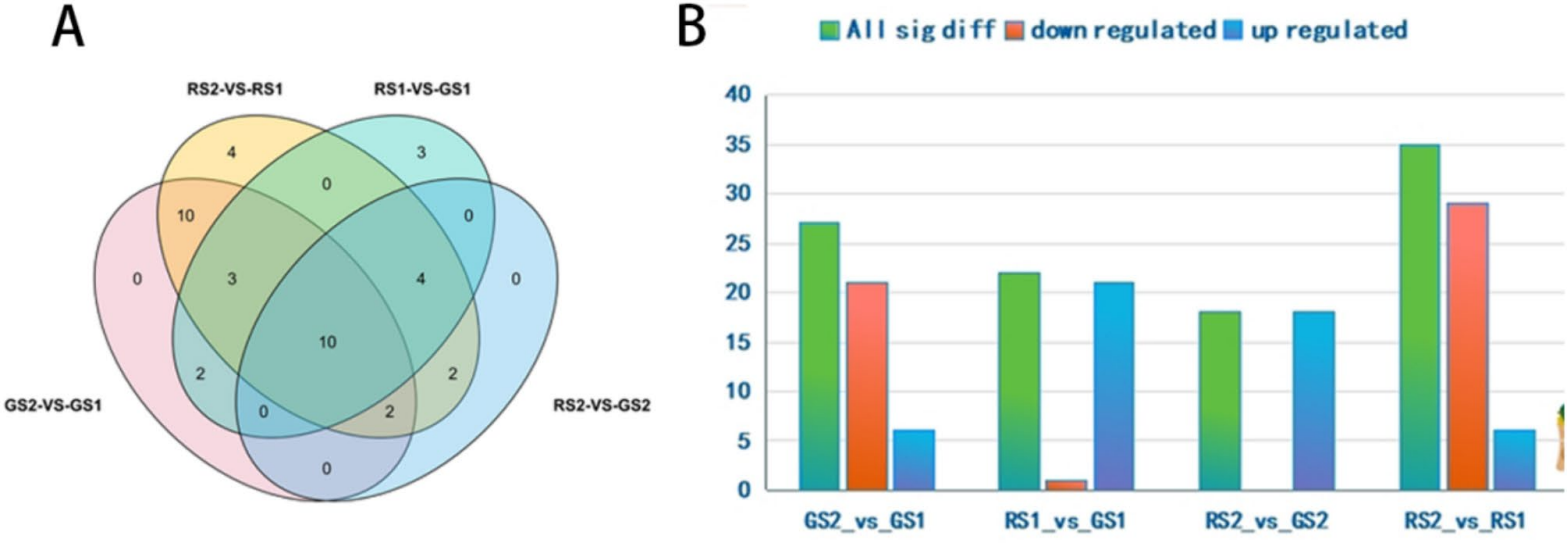
**A** Venn plots of the four grouped differential metabolites; **B** Number of up-and down-regulation of the four grouped differential metabolites

Forty differential carotenoid metabolites were screened in the four groups, including 6 carotenoids (α-carotene, lycopene, ε-carotene, β-carotene, γ-carotene, and phytoalexin) and 34 lutein (hordein, longhornbeam, zeaxanthin, etc.). There were 27, 35, 18, and 22 differential metabolites in the four comparative combinations of GS2 vs. GS1, RS2 vs. RS1, RS2 vs., GS2, and RS1 vs. GS1, respectively (Fig. 5A). Among these, 6 and 21 differential metabolites were upregulated and downregulated, respectively, in the GS2 vs. GS1 combination; 6 and 29 differential metabolites were upregulated and downregulated, respectively, in the RS2 vs. RS1 combination; 18 differential metabolites were upregulated in the RS2 vs. GS2 combination; and 21 and 1 differential metabolites were upregulated and downregulated, respectively, in the GS2 vs. GS1 combination (Fig. 5B).

#### Enrichment analysis of differential carotenoid metabolites

**Fig. 6:**
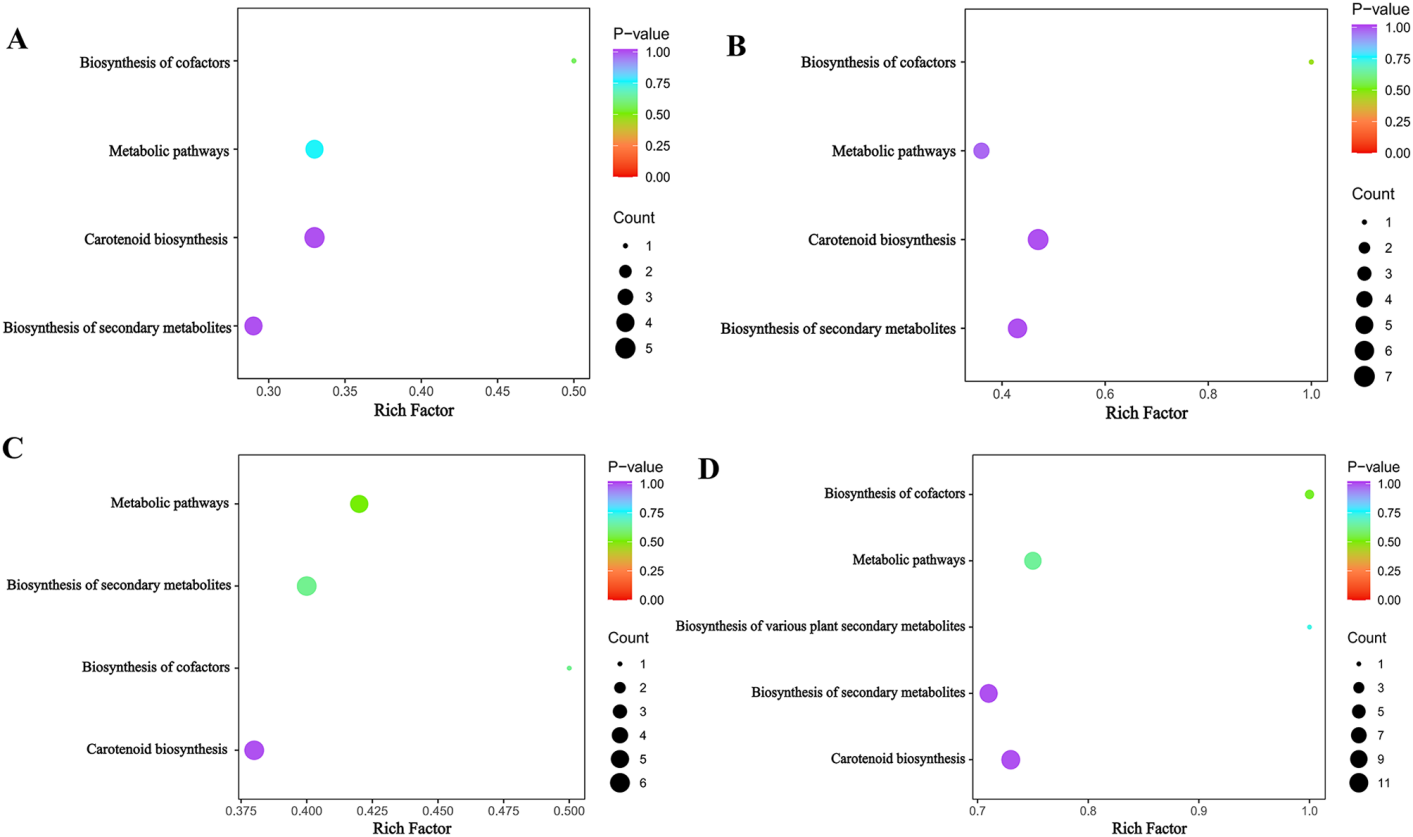
Carotenoid metabolite analysis. (**A-D**) Different enrichment pathways of carotenoid metabolites in RS2 vs. GS2, GS2 vs. GS1, RS1 vs. GS1, and RS2 vs. RS1. Note: The horizontal coordinates indicate the enrichment factors corresponding to each pathway, the vertical coordinates are the pathway names, and the color of the dots is the p-value; the higher the intensity of redness, the more significant is the enrichment. The size of the dots represents the number of different metabolites

In the four comparison groups of GS2 vs. GS1, RS2 vs. RS1, RS2 vs. GS2, and RS1 vs. GS1, the differential carotenoid metabolites were enriched in the ko00906 (carotenoid biosynthesis), ko01100 (metabolic pathway), ko01110 (secondary metabolite biosynthesis), and ko01240 (biosynthesis of cofactors). The highest number of metabolites was enriched in the ko00906 pathway (Fig. 6). The metabolites α-carotene and ε-carotene appeared in all four groups.

### Transcriptome analysis of *C. huana*

#### Quality assessment of the transcriptome sequencing data

To further analyze the molecular mechanisms underlying the metabolic differences between flower colors at the different developmental stages of *C. huana*, four samples were subjected to transcriptome sequencing. The cDNA libraries of the two flower colors of *C. huana* at the different developmental stages were sequenced for the transcriptome by using the Illumina sequencing platform. A total of 630,712,816 filtered sequences were obtained, and 94.6 Gb data were generated. The sequencing quality was assessed, revealing Q20 and Q30 percentages exceeding 97% and 93%, respectively. Additionally, GC content ranged from 44.07 − 45.85%, with an average above 45%.(Supplementary Table 1); these findings indicated the high sequencing quality of this experiment. Thus, this method could be used for subsequent gene expression analysis.

#### Results of transcriptome analysis

**Fig. 7:**
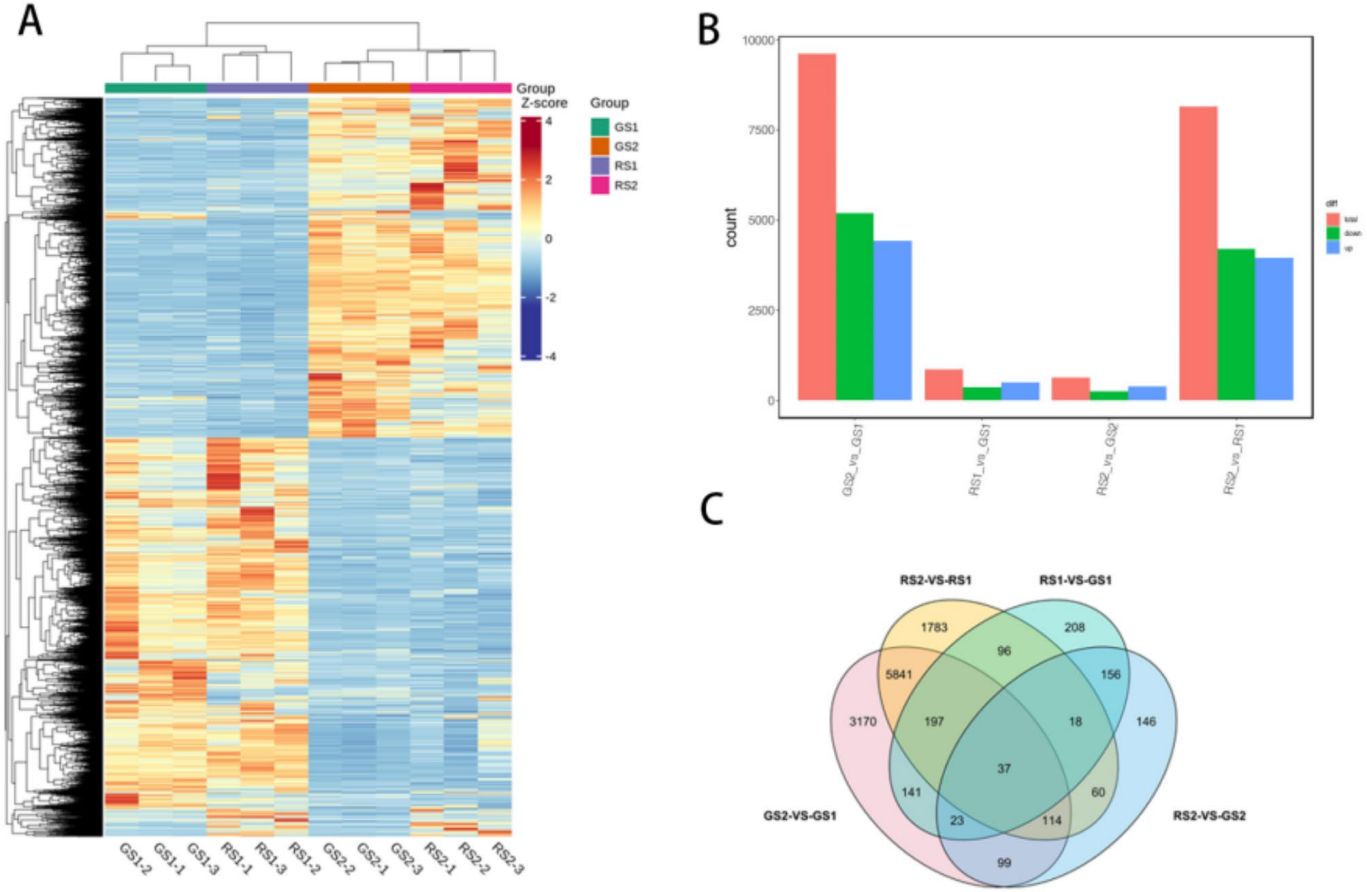
(**A**) Clustering heatmap of the DEGs. (**B**) Histogram of the DEGs. (**C**) Venn diagram of the DEGs

A total of 12,089 DEGs were screened in this study, including 9276 upregulated genes and 10,021 downregulated genes. The criteria were|log2(fold change)| ≥ 1 and false discovery rate ≤ 0.05. FPKM (fragments per kilobase of transcript per million fragments mapped) was used to measure the expression level of transcripts or genes. Subsequently, a hierarchical clustering heatmap was generated using the biological replicates of the three genotypes based on the FPKM values of the DEGs, and the four groups of 12 samples were divided into two major groups, with each biological replicate as a subgroup. The heatmap showed that the early stage of flower development (GS1 and RS1) clustered together and the later flowering stage (GS2 and RS2) clustered together. Furthermore, the heatmap demonstrated that the correlation between the biological replicates in the same sample group was relatively high, thus indicating better replication within the selected sample group(Figure 7A). We identified 9622 significant DEGs in GS2 vs. GS1, of which 4430 genes were upregulated and 5192 genes were downregulated. A total of 876 DEGs were detected in RS1 vs. GS1, with 504 upregulated genes and 372 downregulated genes. In the RS2 vs. GS2 comparison, 653 DEGs were detected, with 391 upregulated genes and 262 downregulated genes. Furthermore, in the RS2 vs. RS1 comparison, 8146 DEGs were found, of which 3951 and 4195 genes were upregulated and downregulated, respectively. The results are shown in Fig. 7(B). The Venn diagram of the DEGs is shown in Fig. 7(C). Thirty-seven genes were commonly expressed in the four groups, thus indicating that these genes were differentially expressed at the different developmental stages. There were fewer DEGs in RS1 vs. GS1 and RS2 vs. GS2 than in GS2 vs. GS1 and RS2 vs. RS1.

#### Analysis of DEGs at the different developmental stages of *C. huana* from Guizhou

To further investigate the biological functions of the obtained DEGs at the different developmental stages of *C. huana*, we performed Gene Ontology (GO) and KEGG pathway enrichment analyses of the DEGs. The GO terms that were significantly enriched for the DEGs in GS1 vs. GS2 were “pectin metabolism process,” “polysaccharide catabolism metabolism process,” “polymerization of cytoskeletal fibers,” and “cell wall macromolecule metabolism process” (Supplementary Fig. 1A). In addition to “polysaccharide catabolic process” and “pectin metabolic process,” the GO terms of the DEGs in RS2 vs. RS1 were enriched in “microtubule” and “galacturonic acid metabolic process.” The GO terms significantly enriched for the DEGs of RS1 vs. GS1 were “carbohydrate transmembrane transporter activity,” “aminoglycan metabolic process,” and “pectin metabolic process.” The GO terms significantly enriched for the DEGs of RS2 vs. GS2 were “phenylpropanol metabolic process,” “lignin metabolic process,” “hydrogen peroxide catabolic process,” and “lignin metabolic process.”

The KEGG pathway enrichment analysis was also performed for the DEGs of the abovementioned four groups. The results showed that the DEGs of the four groups were mainly enriched in “metabolic pathway,” “galactose metabolism,” “secondary metabolite biosynthesis,” and “phenylpropanoid biosynthesis.” Among these genes, TEA014269, TEA031407, TEA023505, TEA006973, TEA013909, TEA030362, and TEA014720 were mainly found in the carotenoid metabolic pathway. TEA005855, novel.7518, TEA029348, TEA009170, TEA004599, novel.10,533, TEA026265, and other genes were mainly found in terpenoid and triterpenoid metabolic pathways.

### Correlation analysis of transcriptomes and metabolomes

#### Integrated analysis of transcriptomics and metabolomics for the VOCs

**Fig. 8:**
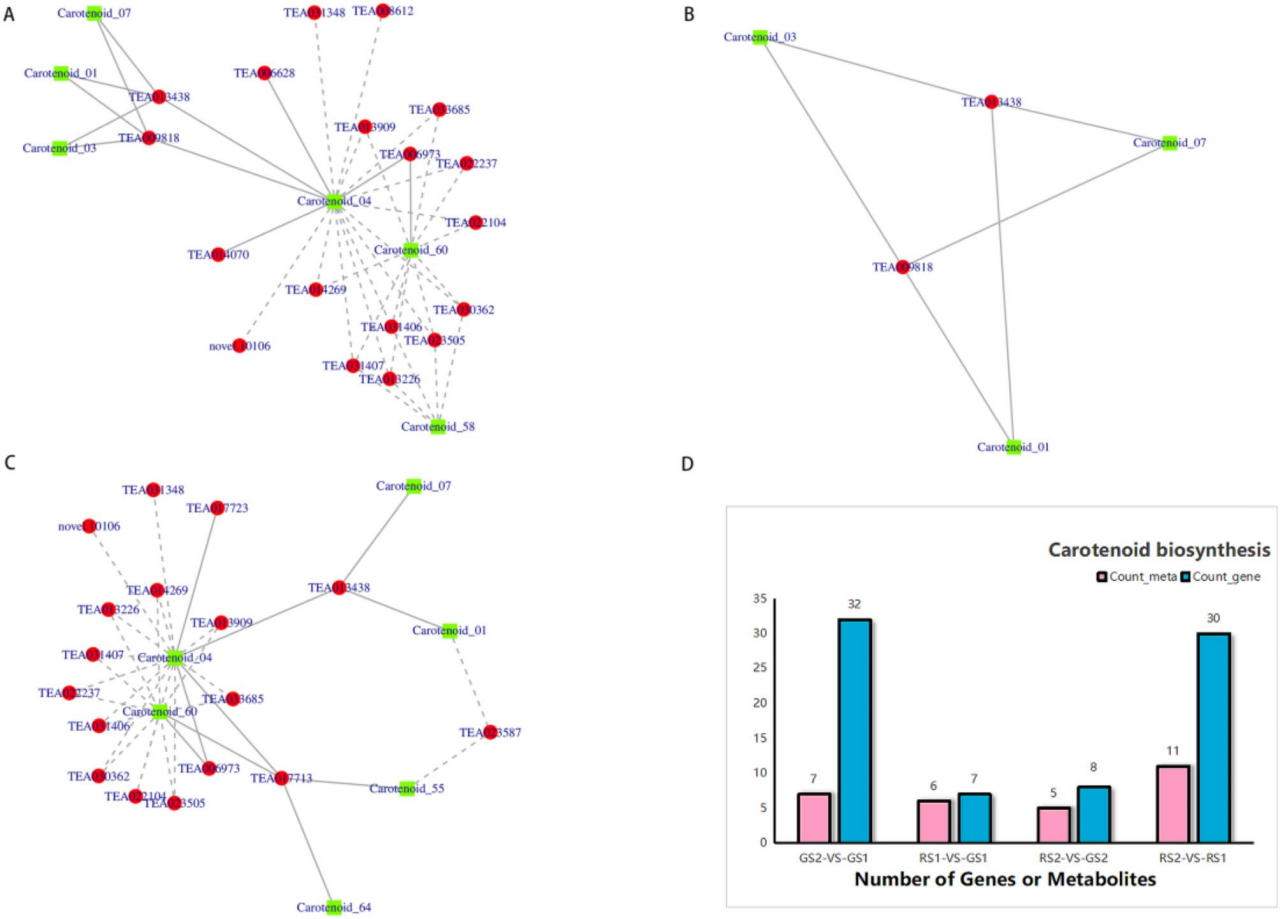
Correlation analysis of the transcriptome and metabolome of *C. huana* flowers. (**A-C**) Interactions between genes and metabolites. (**D**) Pathways co-enriched with genes and metabolites in the GS2 vs. GS1, RS1 vs. GS1, RS2 vs. GS2, and RS2 vs. RS1 groups

We performed Pearson’s correlation analysis to determine the correlation between the differential metabolites and the DEGs during *C. huana* flower development (Pearson’s correlation coefficient > 0.8 or < -0.8, *p* < 0.05). The results showed that 13 genes, including TEA022104, TEA031348, and TEA008612, were upregulated in the GS2 vs. GS1 group. Six metabolites, including lycopene (carotenoid_02), β-cryptoxanthin (carotenoid_60), β-carotene (carotenoid_04), and neoxanthin (carotenoid_58), were downregulated. β-Carotene showed a negative correlation with the genes TEA01771, TEA006973, TEA013438, and TEA017723 and a positive correlation with the genes TEA013909, TEA031348, TEA030362, TEA033685, TEA031406, TEA023505, TEA013226, TEA014269, novel.10,106, and TEA022237 (Figs. 8A-C).

#### Reconstruction of the floral carotenoid biosynthetic metabolic pathway

**Fig. 9:**
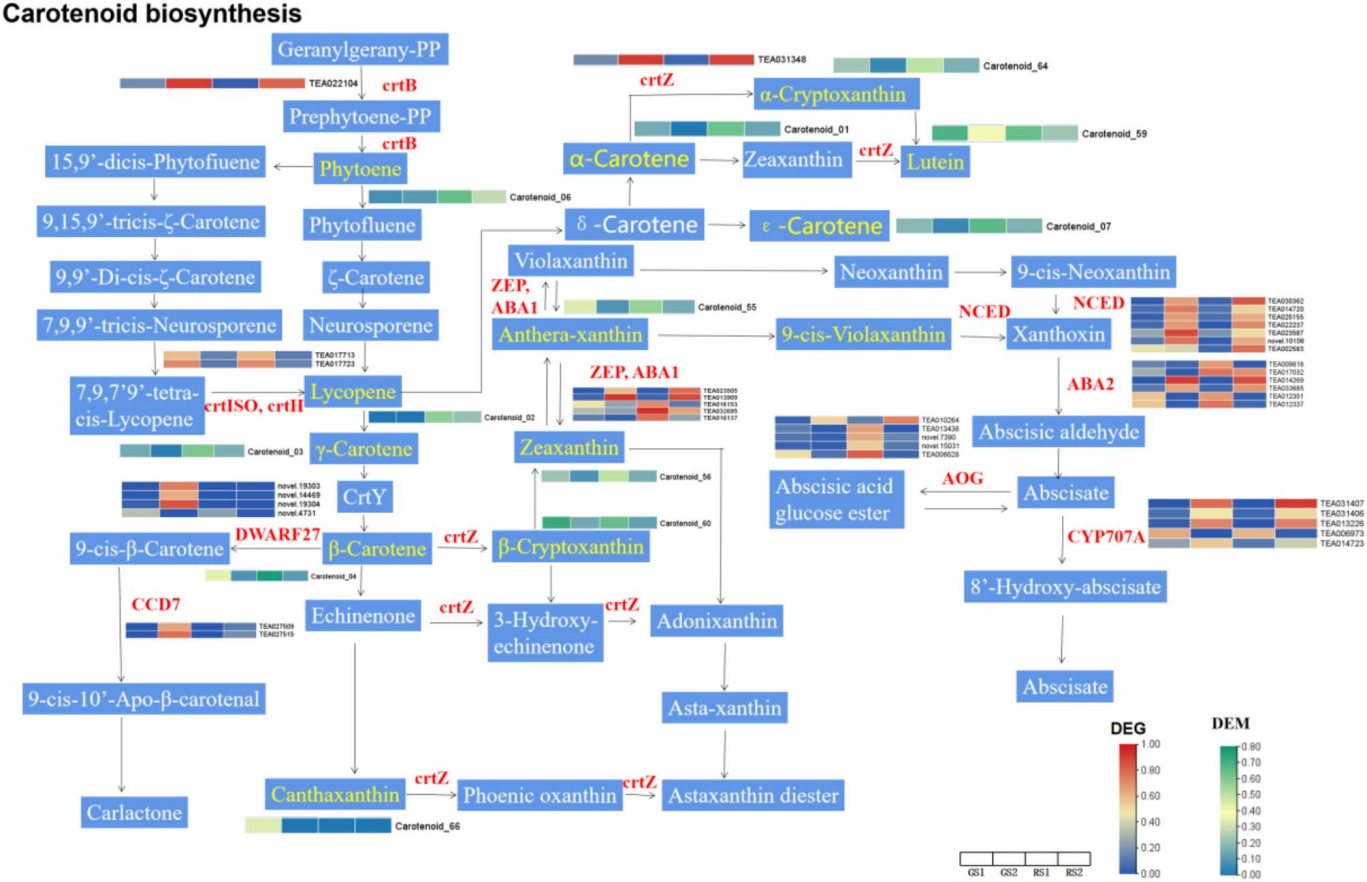
Expression profiles of the DEGs and differentially expressed metabolites associated with the carotenoid biosynthesis pathway. Red letters indicate genes encoding pathway enzymes, and heatmaps are the normalized average values for the two developmental stages in all four samples

Next, we plotted the enrichment histograms based on the KEGG pathway enrichment analysis of the transcriptome and metabolome (Fig. 8D). ko00906 (carotenoid biosynthesis) was significantly enriched in all the four abovementioned combinations. Twelve differential metabolites were enriched in the KEGG pathway map, including α-carotene (carotenoid_01), lycopene (carotenoid_02), γ-carotene (carotenoid_03), β-carotene (carotenoid_04), porphyrin (carotenoid_06), epsilonocarotene (carotenoid_07), phloroglucinolide (carotenoid_55), zeaxanthin (carotenoid_56), lutein (carotenoid_59), and β- Cryptoxanthin(carotenoid_60) Furthermore, 43 DEGs were enriched in the KEGG pathway map, including *crtB*, *crtiSO*, *crtH*, *DWARF27*, *CCD 7*, *CrtZ*, *ZEP*, *ABA1*, *NCED*, *ABA2*, *AOG*, and *CYP707A*. The results of the integrative pathway analysis indicated that the differential metabolites and the DEGs involved in carotenoid biosynthesis showed different accumulation patterns at different developmental periods; moreover, some DEGs showed the same change trends for the downstream metabolites. In the carotenoid biosynthesis pathway, the expression levels of the DEGs *crtB*, *crtZ*, *CCD 7*, *NCED*, *ABA2*, *ZEP*, *ABA1*, and *CYP707A* were higher in GS2 and RS2 than in GS1 and RS1. The gene *DWARF27* exhibited a higher expression level in GS2 than in the other periods. The expression level of the *AOG* gene varied in different samples, with the highest expression in RS2. The metabolites α-carotene, γ-carotene, β-carotene, phytochrome, and ε-carotene showed higher expression levels in RS1 than in the other periods. Lycopene showed a higher expression level in RS1 and RS2 than in GS1 and GS2, and the metabolites lutein, β-cryptoxanthin, and α-cryptoxanthin exhibited a higher expression level in GS1 and RS1 than in GS2 and RS2(Fig. 9).

#### Reconstruction of the biosynthetic metabolic pathways of sesquiterpenoids and triterpenoids in *C. huana* flowers

**Fig. 10:**
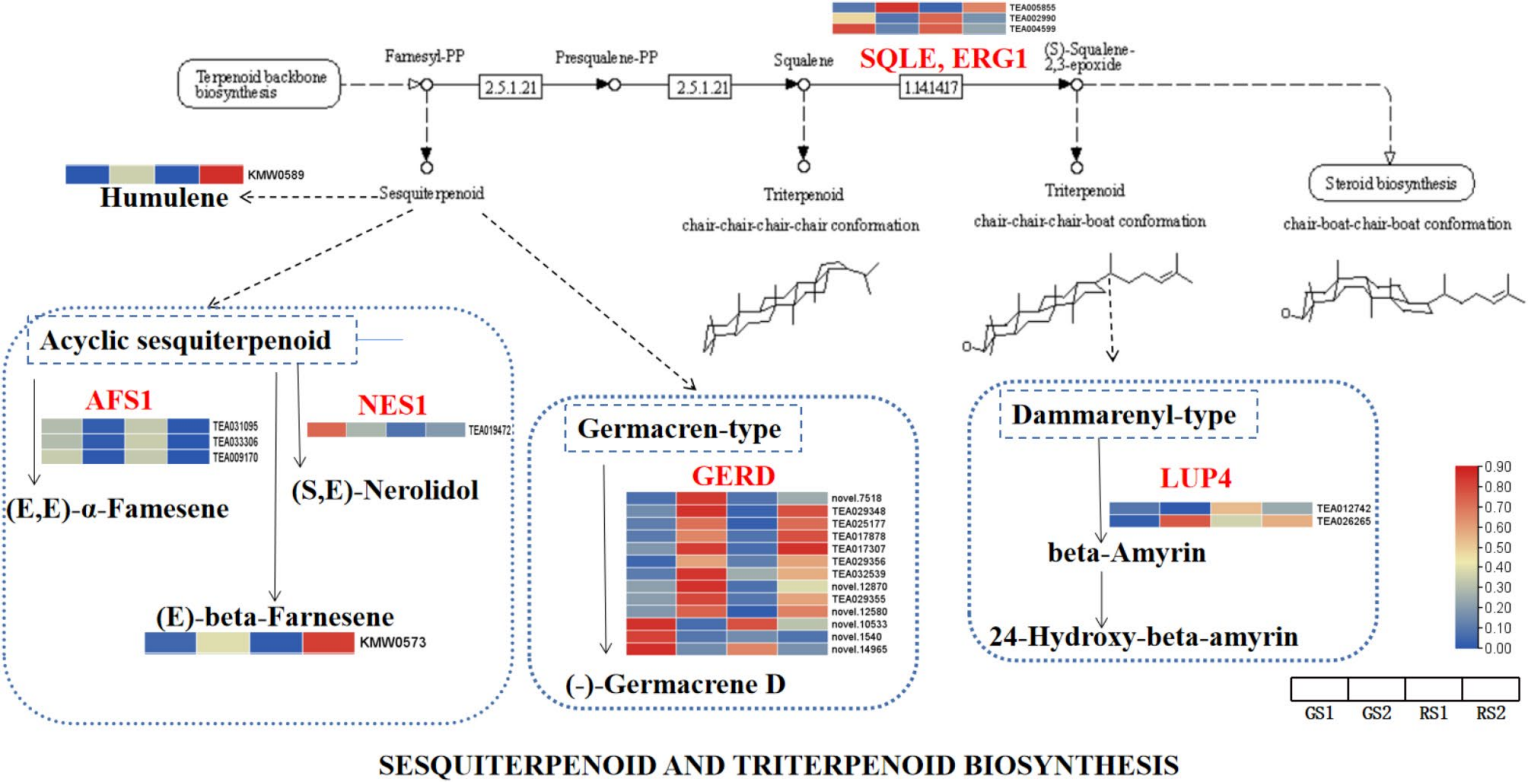
Expression patterns of the genes of the sesquiterpenoid and triterpenoid biosynthetic pathways in *C. huana*

To determine the association between the DEGs and differentially expressed metabolites (DEMs), we conducted the KEGG pathway enrichment analysis on the transcriptomic and metabolomic data. As shown previously, terpenoids are the largest class of plant volatiles. The metabolites (E)-β-famesene (KMW0589) and humulene (KMW0573) were significantly enriched in the KEGG pathway analysis. The 22 DEGs with significant enrichment in the KEGG pathway analysis included *SQLE*, *ERG 1*, *AFS 1*, *NES 1*, *GERD*, and *LUP 4*. The expression of (E)-β-famesene and humulene was higher in GS2 and RS2 than in GS1 and RS1; however, the expression of these metabolites was lower in RS2 than in GS1. Furthermore, the DEGs *SQLE*, *ERG 1*, and *AFS 1* showed higher expression in GS1, RS1, and GS2, and the *GERD* gene showed a higher expression level in GS2 and RS2 than in GS1 and RS1. The *NES 1* gene showed the highest expression in GS1, and the *LUP 4* gene showed a lower expression in GS1 than in the other stages(Fig. 10).

## Conclusions

The composition and content of different metabolites induce changes in flower traits during *C. huana* growth and development. The integrated metabolome and transcriptome analyses indicated a significant correlation between the expression of carotenoid and terpenoid metabolites and related DEGs. These related DEGs play an important role in the floral development of *C. huana*.The results showed that *C. huana* is rich in volatile compounds. A total of 372 metabolites were detected in *C. huana*, which mainly included 72 terpenoids, 67 heterocyclic compounds, nitrogen-containing compounds, esters, aromatic hydrocarbons, 39 alcohols, and others; terpenoids, heterocyclic compounds, and esters were the predominant volatiles. Forty carotenoids were identified by carotenoid content analysis, and 10 genes were involved in carotenoid biosynthesis were screened for their significant differential expression, namely 15-cis-phytoene synthase (*crtB*), prolycopene isomerase (*crtISO*, *crtH*), beta-carotene 3-hydroxylase (*crtZ*), beta-carotene isomerase (*DWARF27*), 9-cis-beta-carotene 9ʹ,10ʹ-cleaving dioxygenase (*CCD7*), zeaxanthin epoxidase (*ZEP*, *ABA1*), 9-cis-epoxycarotenoid dioxygenase (*NCED*), xanthoxin dehydrogenase (*ABA2*), abscisate beta-glucosyltransferase (*AOG*), and (+)-abscisic acid 8ʹ-hydroxylase (*CYP707A*). A total of 12,089 differential genes were screened by transcriptome analysis.The results of this study enriched the transcriptome data and provided new insights into the mechanisms of color and odor formation in the flowers of *C. huana*.

## Electronic supplementary material

Below is the link to the electronic supplementary material.

Supplementary Material 1

## Data Availability

All data generated or analysed during this study are included in this published article [and its supplementary information files].
