# Supplementary material for: Integrated transcriptomics and metabolomics analysis of volatiles and variations in carotenoid biosynthesis during different developmental stages of Camellia huana
Source: BMC Plant Biol. 2025 May 31;25:734. doi: 10.1186/s12870-025-06549-z (PMC12125916; doi:10.1186/s12870-025-06549-z)
Supplement: Supplementary file 1 — Supplementary Material 1 [file 12870_2025_6549_MOESM1_ESM.pdf]

一、Supplementary Figure 1

Supplementary Figure. 1

The TIC diagram of the mixed sample of the left golden flower tea and the TIC diagram of the right second QC sample

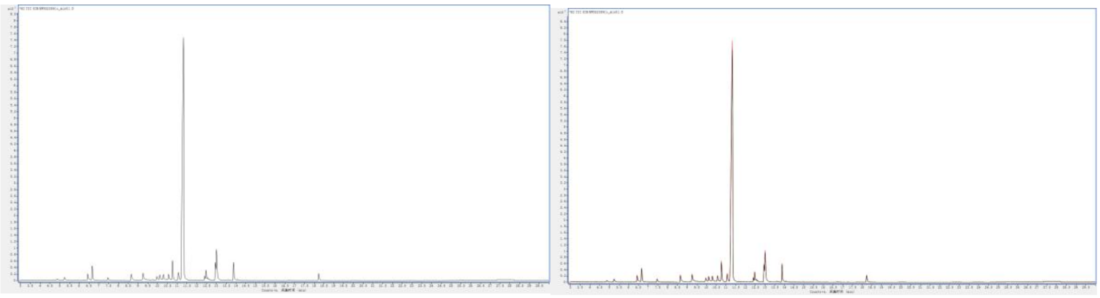

二、Supplementary Figure 1 (GO enrichment analysis)

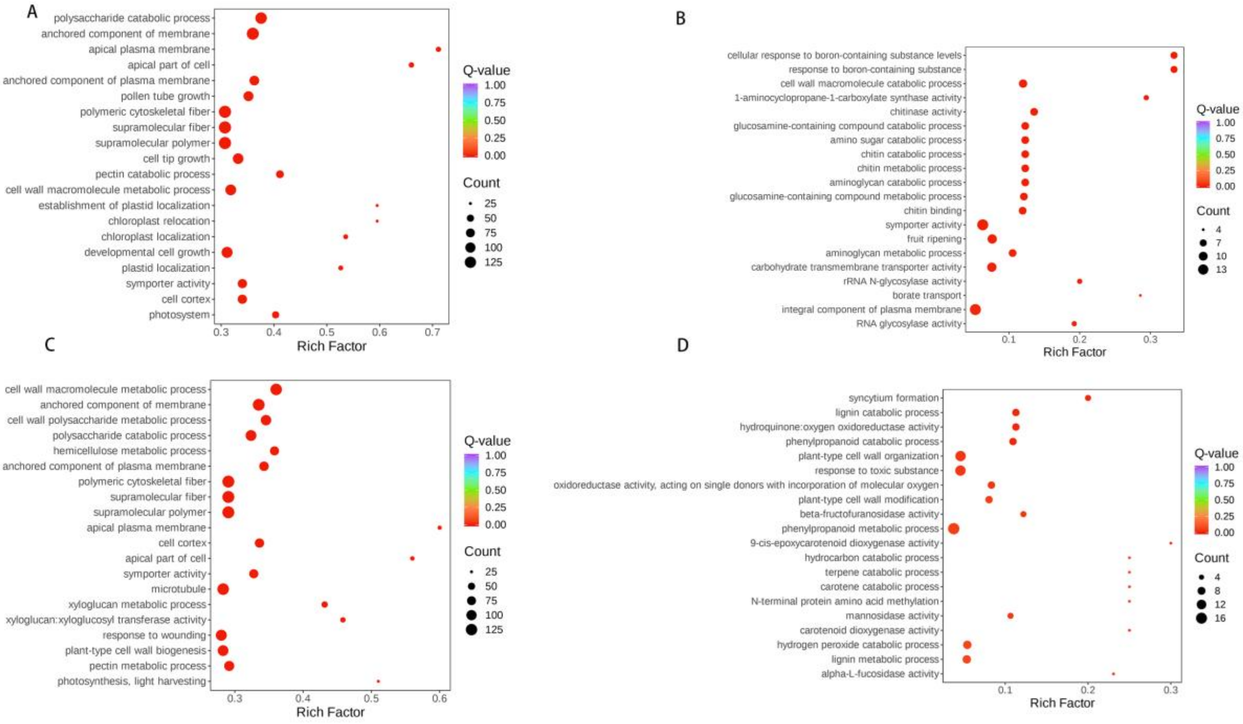

Supplementary Figure 1. DEGs enriched on different GO terms A、GO terms of DEGs in GS2-vs-GS1 B、GO terms of DEGs in RS1-vs-GS1 C、GO terms of DEGs in RS2-vs-RS1 D、GO terms of DEGs in RS2-vs-GS2

三、Supplementary Table 1

Schedule 1 Significant differential metabolites of GS2-VS-GS1

Type	Class I	Compound	VIP	P-value	Fold_Change
up	Alcohol	1-Heptanol	1.72E+00	2.50E-02	1.77E+01
		3-Mercapto-3-methylbutanol	1.72E+00	2.23E-02	1.20E+01
		1-Hexanol, 2-ethyl-	1.62E+00	7.10E-03	2.02E+00
	Terpenoids	(E)-.beta.-Famesene	1.68E+00	2.38E-03	4.34E+00

		Humulene	1.73E+00	8.96E-03	#DIV/0!
		7-Oxabicyclo[2.2.1]heptane, 1-methyl-4-(1-methylethyl)-	1.72E+00	2.88E-04	7.61E+00
		2H-Pyran, 3,6-dihydro-4-methyl-2-(2-methyl-1-propenyl)-	1.69E+00	6.01E-04	3.10E+00
		5,7-Octadien-4-one, 2,6-dimethyl-, (Z)-	1.68E+00	3.21E-04	4.22E+00
	Hydrocarbons	Heptane, 2,2,4,6,6-pentamethyl-	1.71E+00	1.21E-02	3.13E+00
	Ketone	5,9-Undecadien-2-one, 6,10-dimethyl-, (E)-	1.72E+00	1.18E-02	7.96E+00
		5-Methyl-(E)-2-hepten-4-one	1.72E+00	2.11E-02	7.07E+00
	Heterocyclic compound	1,2,4,5-Tetrazin-3-Amine	1.72E+00	1.60E-02	8.80E+00
	Ester	2-Propenoic acid, pentyl ester	1.72E+00	2.13E-02	1.88E+01
down	Heterocyclic compound	2-Propenal, 3-(2-furanyl)-	1.41E+00	2.86E-02	3.55E-01
		Ethanethioic acid, S-(2-furanylmethyl) ester	1.53E+00	5.23E-02	4.80E-01

Schedule 2 Significant differential metabolites of RS2-VS-GS2

Type	Class I	Compound	VIP	P-value	Fold_Change
up	Terpenoids	Cyclohexanol, 1-methyl-4-(1-methylethylidene)-	1.12E+00	1.78E-01	2.47E+00
	Ester	Methyl salicylate	1.21E+00	1.57E-01	3.67E+00
down	Terpenoids	cis-Chrysanthenol	1.65E+00	3.70E-03	0.00E+00
		Cyclohexanone, 5-methyl-2-(1-methylethyl)-, trans-	1.53E+00	5.31E-03	2.73E-01
	Hydrocarbons	Albene	1.52E+00	5.64E-03	2.73E-01
	Ketone	1-Hepten-3-one	1.54E+00	1.89E-02	4.16E-01
	Heterocyclic compound	6-Ethyl-5,6-dihydro-2H-pyran-2-one	1.52E+00	7.44E-03	2.07E-01
		2-Propenal, 3-(2-furanyl)-	1.39E+00	2.18E-02	3.34E-01
		4-Aminopyridine	1.53E+00	5.81E-03	3.14E-01

Schedule 3 Significant differential metabolites of RS1-VS-GS1

Type	Class I	Compound	VIP	P-value	Fold_Change
up	Alcohol	p-Cymen-7-ol	1.62E+00	1.85E-01	1.24E+01
	Aromatics	2-Methoxy-4-vinylphenol	1.21E+00	3.34E-01	3.74E+00
	Phenol	Phenol, 4-(1,1-dimethylpropyl)-	1.64E+00	1.12E-01	3.09E+00
	Terpenoids	Humulene	1.72E+00	2.10E-02	2.42E+00
	Ketone	5,9-Undecadien-2-one, 6,10-dimethyl-, (E)-	1.68E+00	3.90E-02	2.25E+00
		2-Cyclopenten-1-one, 3-methyl-2-(2-pentenyl)-, (Z)-	1.72E+00	4.20E-02	2.51E+00
		2-Butanone,	1.73E+00	3.82E-02	2.64E+00

		4-(2,6,6-trimethyl-2-cyclohexen-1-yl)-			
	Heterocyclic compound	Ethanethioic acid, S-(2-furanylmethyl) ester	1.65E+00	4.43E-02	2.29E+00
	Ester	1,2-Ethanediol, monobenzoate	1.48E+00	2.31E-01	4.04E+00
		Methyl salicylate	1.54E+00	9.04E-02	2.59E+00
down	Alcohol	1-Heptanol	1.69E+00	4.83E-02	3.87E-01
		3-Mercapto-3-methylbutanol	1.67E+00	3.93E-02	4.11E-01
	Terpenoids	Cyclohexanone, 5-methyl-2-(1-methylethyl)-, trans-	1.38E+00	8.45E-02	4.44E-01
		5,7-Octadien-4-one, 2,6-dimethyl-, (Z)-	1.62E+00	1.55E-02	4.76E-01
	Hydrocarbons	Albene	1.38E+00	7.94E-02	4.33E-01
	Ketone	5-Methyl-(E)-2-hepten-4-one	1.69E+00	3.50E-02	4.28E-01
	Heterocyclic compound	6-Ethyl-5,6-dihydro-2H-pyran-2-one	1.30E+00	9.10E-02	3.95E-01
		1,2,4,5-Tetrazin-3-Amine	1.71E+00	2.40E-02	4.07E-01
		4-Aminopyridine	1.37E+00	8.87E-02	4.76E-01
	Ester	2-Propenoic acid, pentyl ester	1.70E+00	3.61E-02	3.66E-01

Schedule 4 Significantly different metabolites of RS2-VS-RS1

Type	Class I		VIP	P-value	Fold_Change
up	Alcohol	1-Heptanol	1.45E+00	1.04E-02	6.41E+00
		p-Cymen-7-ol	1.03E+00	2.39E-01	4.08E+00
		3-Mercapto-3-methylbutanol	1.47E+00	1.80E-02	5.60E+00
		1-Hexanol, 2-ethyl-	1.33E+00	3.43E-02	2.04E+00
	Terpenoids	(E)-.beta.-Famesene	1.49E+00	1.69E-02	1.21E+01
		Humulene	1.51E+00	1.12E-02	#DIV/0!
		1-methyl-4-(1-methylethyl)-	1.46E+00	6.70E-02	1.21E+01
		cis-Chrysanthenol	1.42E+00	7.94E-02	#DIV/0!
	Hydrocarbons	Tetradecane, 4-methyl-	1.47E+00	2.18E-03	2.89E+00
		Undecane, 2-methyl-	1.43E+00	1.10E-02	2.01E+00
		Heptane, 2,2,4,6,6-pentamethyl-	1.51E+00	2.36E-03	3.17E+00
	Ketone	5,9-Undecadien-2-one, 6,10-dimethyl-, (E)-	1.50E+00	1.81E-02	1.74E+01
		5-Methyl-(E)-2-hepten-4-one	1.48E+00	1.77E-02	3.27E+00
	Heterocyclic compound	1,2,4,5-Tetrazin-3-Amine	1.47E+00	1.42E-02	4.49E+00
	Ester	2-Propenoic acid, pentyl ester	1.46E+00	1.67E-02	6.76E+00
down	Terpenoids	3-Cyclohexen-1-ol, 1-methyl-4-(1-methylethyl)-	1.30E+00	8.31E-02	4.70E-01
